# Gut-Lung Crosstalk in Sepsis-Induced Acute Lung Injury

**DOI:** 10.3389/fmicb.2021.779620

**Published:** 2021-12-23

**Authors:** Xin Zhou, Youxia Liao

**Affiliations:** Department of ICU/Emergency, Wuhan University, Wuhan Third Hospital, Wuhan, China

**Keywords:** sepsis, acute lung injury, gut-lung crosstalk, inflammation, gut microbiome

## Abstract

Acute lung injury (ALI) and acute respiratory distress syndrome (ARDS) are common acute and severe cases of the respiratory system with complicated pathogenesis and high mortality. Sepsis is the leading indirect cause of ALI/ARDS in the intensive care unit (ICU). The pathogenesis of septic ALI/ARDS is complex and multifactorial. In the development of sepsis, the disruption of the intestinal barrier function, the alteration of gut microbiota, and the translocation of the intestinal microbiome can lead to systemic and local inflammatory responses, which further alter the immune homeostasis in the systemic environment. Disruption of homeostasis may promote and propagate septic ALI/ARDS. In turn, when ALI occurs, elevated levels of inflammatory cytokines and the shift of the lung microbiome may lead to the dysregulation of the intestinal microbiome and the disruption of the intestinal mucosal barrier. Thus, the interaction between the lung and the gut can initiate and potentiate sepsis-induced ALI/ARDS. The gut–lung crosstalk may be a promising potential target for intervention. This article reviews the underlying mechanism of gut-lung crosstalk in septic ALI/ARDS.

## Introduction

Acute lung injury (ALI)/acute respiratory distress syndrome (ARDS) is a life-threatening condition of respiratory failure (Matthay et al., 2019). ALI/ARDS is a common clinical critical disease with rapid onset and high mortality and one of the primary causes of death in critically ill patients (Griffiths et al., 2019). According to statistics, more than three million ARDS patients are diagnosed worldwide every year, accounting for 10% of the number of people hospitalized in intensive care units (ICU) (Abe et al., 2018). The treatment of ALI/ARDS remains elusive. Despite major recent advances in the supporting care for ARDS, including the use of extracorporeal membrane oxygenation (ECMO), protective lung ventilation maneuvers, and statins (Fan et al., 2018), the mortality from ARDS is still high (34.9–46.1%) (Fernando et al., 2021). Sepsis is the leading indirect cause of ALI/ARDS in the ICU (Bellani et al., 2016), and the lung is the first affected and the most vulnerable organ during sepsis (Fan and Fan, 2018). Direct sepsis-induced ALI/ARDS arises from pulmonary infections, and indirect sepsis-induced ALI/ARDS arises from extrapulmonary infections (Bellani et al., 2016). It is worth noting that the mortality rate of ARDS caused by sepsis is higher than that of ARDS caused by other factors (Chen et al., 2019). Although the biology underlying sepsis-induced ALI/ARDS is complicated and multifactorial, our current understanding involves the release of inflammatory cytokines and the disruption of the lung microvascular barrier (Huppert et al., 2019).

Severe acute inflammation plays a crucial role in septic ALI/ARDS (Lelubre and Vincent, 2018). Pathogens activate the innate immune response of epithelial cells and alveolar macrophages, followed by migration and aggregation of neutrophils and monocytes, release of the inflammatory cytokines TNF-α, IL-1β, and IL-6, loss of alveolar-capillary barrier integrity, and increased permeability, leading to sepsis-associated ALI/ARDS (Luyt et al., 2020).

The gut microbiome coexists harmoniously with the host and plays multifarious beneficial roles in the body, such as shaping the immune system, maintaining homeostasis, and others (Morais et al., 2021). An increasing body of evidence has illustrated the role of the gut microbiome in the occurrence, development, and outcomes of sepsis (Adelman et al., 2020). Sepsis induces significantly compromised gut barrier integrity (Yoseph et al., 2016), which allows intact microbes and microbiota products to translocate, resulting in amplification of the systemic inflammatory response and contribution to multiple organ failure (Dickson, 2016). This imbalanced interaction between the gut barrier, immune system, endogenous microorganisms, and lung may lead to the deterioration of the systemic inflammatory response and the potentiation of ALI/ARDS. Hence, a better understanding of gut-lung crosstalk in sepsis-related ALI/ARDS may contribute to clarifying this complex disease and laying the foundation for new treatments.

### Effect of Septic Acute Lung Injury/Acute Respiratory Distress Syndrome on the Gut

**FIGURE 1:**
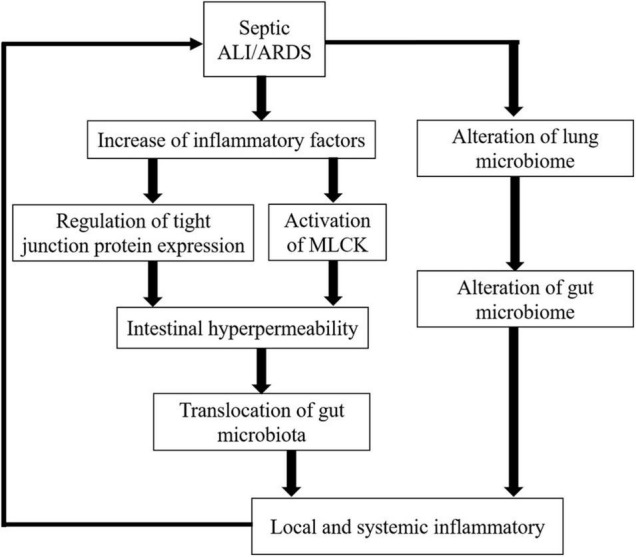
Effect of increased inflammatory cytokines and lung microbiome alteration on the gut in septic ALI/ARDS.

During sepsis, the onset of ALI/ARDS is related to the activation of a complex inflammatory cytokine cascade. The continuous recruitment of inflammatory cells perpetuates a vicious cycle that produces more pro-inflammatory cytokines, which interact with and influence each other to promote severe damage to the alveolar-capillary membrane as well as respiratory failure. Moreover, researchers have broadened the scope of septic ALI/ARDS to another key factor: the lung microbiome. In septic-ALI/ARDS, the inactivation of the bactericidal layer of the alveolar epithelium (Li et al., 2019), the flow of protein-rich alveolar exudate, the establishment of oxygen gradients, the surge of inflammatory mediators, and the impairment of local immune defenses (Dickson et al., 2015) may alter the lung microbiome that in turn perpetuates a positive-feedback loop of inflammation, injury, and further dysbiosis (Dickson et al., 2014). Along with an increase in inflammatory molecules and alteration of the lung flora, septic-ALI/ARDS is responsible for gut microbiota imbalance and gut barrier disruption (Figure 1).

#### Intestinal Barrier Disruption by Increased Cytokines During Sepsis

**FIGURE 2:**
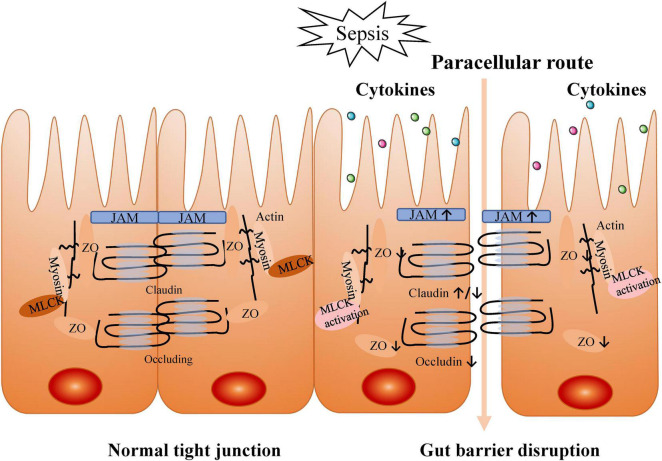
Intestinal barrier disruption by increased cytokines during sepsis. Tight junctions between cells play a critical role in gut barrier function. In sepsis, tight junctions are destructed by inflammatory cytokines production.

During septic ALI/ARDS, increased cytokine levels may cause impairment of the intestinal barrier. This selective barrier is composed of intestinal mucosal epithelial cells and inter-cell connections. When the selective barrier functions normally, it allows the cell-side movement of water, solutes, and immunomodulatory factors but prevents the movement of macromolecules and microorganisms (Weström et al., 2020). Tight junctions between cells play a critical role in the gut barrier function, and the cells are connected to the intracellular cytoskeleton by tight junction proteins [e.g., occludin, claudin family, junctional adhesion molecules (JAM), myosin light chain, zonula occludens (ZO); Krug and Fromm, 2020]. The increase of inflammatory cytokines during sepsis results in intestinal hyperpermeability through the up-regulation of claudin 2 and JAM and down-regulation of claudin 5 and ZO-1 (Yoseph et al., 2016). Alternatively, sepsis can also redistribute claudins 1, 3, 4, 5, and 8, resulting in intestinal barrier dysfunction (Li et al., 2009). Claudins are a protein family of up to 27 members in mammals. Expression of claudins 1–19 has been examined throughout the rat and mouse intestine and that of claudins 20–24 in the mouse upper small intestine. Members of the claudin family are major driving forces in the formation of paracellular barriers (Günzel and Yu, 2013). Claudins can be roughly divided into sealing or pore-forming claudins. Alteration of claudin expression may result in a decreased or increased paracellular transport of solutes as well as an increased permeability to macromolecules (Günzel, 2017). To date, claudin-2, -10b, and -15 qualify as cation pores and claudin-10a and -17 as anion pores, which are both acknowledged as pore-forming claudins (Odenwald and Turner, 2013). Therefore, an increase in a pore-forming protein, such as claudin-2, would directly cause hyperpermeability and deterioration of the barrier function, while a decrease in a sealing protein, such as claudin-5, would lead to the same result in a mechanistically complementary manner. Beyond that, the activation of myosin light chain kinase (MLCK) by inflammatory cytokines (TNF-α, IL-6, and IL-1β) is also associated with paracellular hyperpermeability. Cytokines further activated MLCK in a feed-forward mechanism, partly via altering ZO-1 and claudin 15 (Lorentz et al., 2017). MLCK phosphorylates the myosin regulatory light chain, which leads to contraction of the actin-myosin ring, increasing intestinal permeability (Su et al., 2020). Indeed, the significant increase of the intestinal permeability is closely interrelated to the occurrence and development of sepsis (Assimakopoulos et al., 2018), such as sepsis secondary intratracheal instillation of Pseudomonas aeruginosa due to gut mucosal and microvascular injury and gut barrier dysfunction (Yu and Martin, 2000). Recent studies have reported that gut microbes, represented by Bacteroidetes and Enterobacteriaceae, translocate across the intestinal mucosa and even enter the lung in sepsis and ARDS patients (Dickson et al., 2016; Panzer et al., 2018). However, there is no direct evidence that gut-derived bacteria or bacterial products, such as endotoxin, are present in the blood of septic patients (Haussner et al., 2019). Studies have suggested that ligation of mesenteric lymphatic vessels attenuates lung injury and neutrophil activation and improves survival in mice with endotoxemia (Badami et al., 2008). Moreover, mesenteric lymphatic vessels collected from critically ill mice induces lung injury upon intravenous injection in healthy mice (Senthil et al., 2007). Thus, translocation via intestinal lymphatic vessels is regarded (Haussner et al., 2019), which is called gut–lymph hypothesis. In detail, bacterial translocation gives rise to a local activation of the mucosal immune system (MIS). MIS stimulates the production of inflammatory substances that enter the lung and systemic circulation through the mesenteric lymphatics. These danger-associated molecular patterns (DAMPs) are recognized by innate immune cells to promote pro-inflammatory pathways. A massive release of DAMPs accelerates the progression of organ damage and multiple organ dysfunction syndrome (MODS), further aggravating gut injury, forming a vicious cycle (Deitch, 2012; Assimakopoulos et al., 2018; Haussner et al., 2019). Therefore, the destruction of the gut barrier contributes to the translocation of gut microbes and/or their products to the systemic circulation via the mesenteric lymphatics, exacerbating significant host inflammation and acute pulmonary edema with a positive feedback (Figure 2).

Notably, toll-like receptor 4 (TLR4) is widely expressed in intestinal stem cells and regulates their proliferation or apoptosis (Chen et al., 2018). During sepsis, increased cytokine levels inhibit intestinal cell regeneration and promote apoptosis in a TLR4-dependent manner (Mazmanian et al., 2008). At the cellular level, crypt cell proliferation is markedly decreased (Coopersmith et al., 2003), with increased intestinal epithelial cells (IECs; Coopersmith et al., 2002) and crypt cell apoptosis (Perrone et al., 2012) following sepsis. Although IECs migrate in a TLR-4-dependent manner (Neal et al., 2013), changes in IEC proliferation and apoptosis far exceed this slow course, leading to a shorter villi length (Dominguez et al., 2011). Moreover, cytokines cause an abnormal intestinal and barrier function of the mucous layer, characterized by reduced thickness, diminished lumen coverage, and poor adhesion (Chang et al., 2012). All of these factors further decrease the effectiveness of the gut barrier.

All in all, an increase of inflammatory cytokines induces gut barrier dysfunction, intestinal hyperpermeability, bacterial translocation, and amplification of the inflammatory responses. Subsequently, this expansion of the systemic inflammatory response contributes to lung injury.

#### Role of Lung Dysbiosis in the Gut–Lung Axis

Sepsis-associated ALI/ARDS results in the alteration of lung microbiota both in the mice model and in patients. The etiology of pulmonary dysbiosis in sepsis patients is complex and includes endogenous (e.g., hypoxia and ischemia-reperfusion injury) and external factors (e.g., endotracheal intubation, mechanical lung ventilation, inhaled oxygen, and antibiotics). Poroyko et al. (2015) showed in an ALI mouse model induced by the intratracheal instillation of lipopolysaccharide (LPS) that the bacterial DNA burden in bronchoalveolar lavage (BAL) was increased fivefold, whereas the community complexity measured by the Shannon diversity index was significantly decreased. The major trend in the microbial community reaction to LPS-induced ALI was the loss of Firmicutes, represented by Alicyclobacillaceae, and the bloom of Proteobacteria, represented by Brucellaceae and Xanthomonadaceae. Dickson et al. (2016) also found that the community richness was also higher in a mouse model of sepsis induced by caecal ligation and puncture (CLP). The lower respiratory tract was rapidly enriched with bacteria in the gastrointestinal tract, including members of the Bacteroidales order, Enterococcus species, and Lachnospiraceae species, and remained in this status for 5 days. Furthermore, they sequenced lung microbiota of mice exposed to intratracheal LPS to model direct lung injury and observed enrichment of Enterobacteriaceae-classified OTU in post-sepsis lungs. Based on the above experimental evidence, they further analyzed bacterial communities from the BAL fluid of 68 patients with ARDS. The gut-associated Bacteroides OTU, the most abundant genus in the human gut microbiome, were common in the lungs of ARDS patients (41%) but absent in the lungs of the healthy controls. Furthermore, the gut-associated Bacteroides OTU are most strongly correlated with the severity of systemic and alveolar inflammation. Another study conducted by Dickson reconfirmed prior findings (Dickson et al., 2020). They found the bacterial load was greater in patients with ARDS. In addition, they compared the lung bacterial community composition in BAL specimens from ARDS and non-ARDS patients. The predominant clusters in non-ARDS patients were Streptococcaceae, Veillonellaceae, Prevotellaceae, Verrucomicrobiaceae, and Flavobacteriaceae, which are common in healthy lungs and negative sequence control samples. However, in ARDS patients, the bacterial community in the lungs was characterized by Pasteurellaceae and Enterobacteriaceae. Similar to their prior results, gut-derived Enterobacteriaceae are also correlated with the ARDS status. Notably, the Enterobacteriaceae taxonomy was highly aligned with the ARDS-associated bacterial taxon, as identified by Panzer et al. (2018). They also proved that the microbiome is associated with ARDS development. A recent clinical study (Schmitt et al., 2020) compared the pulmonary microbiota in 15 patients with sepsis-induced ARDS undergoing abdominal surgery and 15 patients post esophagectomy. In the ARDS group, the α-diversity index of the pulmonary microbiome was significantly decreased, which was related to the length of the ICU stay and the need for ventilator use, suggesting that alteration of the lung microbiome may represent a mechanism of pathogenesis in septic ALI/ARDS.

Changes in the lung flora may affect the composition of the gut flora in sepsis-induced ALI/ARDS. One previous study manifested that lung microbiota imbalance in a sepsis-related ALI murine model increased the total bacterial count in the cecum (Sze et al., 2014). In clinical cases, the most common source of sepsis is the lung (Wang et al., 2013). Rosa et al. (2020) found that Vancomycin treatment of acute Pseudomonas aeruginosa pneumonia in mice can induce intestinal dysbacteriosis, resulting in an increase in the number of Proteus, a decrease in the number of bacteroides, and inflammatory changes in the intestinal tract. After fecal microbiota transplantation, the susceptible phenotype and tissue injury phenotype were reversed in mice. Moreover, the pulmonary allergic response also influences the composition of the intestinal microbiota (Vital et al., 2015). In the context of experimental influenza infection, it has been reported that the IFN-γ produced by lung-derived CCR9 + CD4 + T cells changed the composition of the gut microbiota, and caused intestinal immune injury (Wang et al., 2014). Moreover, the pulmonary production of IFN-Is promotes the consumption of obligate anaerobic bacteria and the enrichment of proteobacteria in the gut, leading to significant intestinal dysregulation (Deriu et al., 2016). Thus, lung inflammation directly changes the intestinal bacterial community structure and further worsens lung inflammation (Vital et al., 2015). However, only very limited data have been reported on how long dysbiosis in sepsis-induced ALI/ARDS causes gut dysbiosis. The alveolo-capillary membrane becomes increasingly permeable in sepsis-induced ALI/ARDS as a result of a direct (primarily epithelial) or indirect (primarily endothelial) injury. It is reasonable to posit that alveolo-capillary permeability might be at the highest risk of gut–lung bacterial translocation. Considerable efforts are needed to increase our knowledge about the influence of post-sepsis lung injury on gut microbiota.

### Effect of Gut Microbiome on Gut-Lung Crosstalk

#### Role of Gut Microbiota on the Sepsis

The human gastrointestinal tract is the harbor of 100 trillion bacteria, which are ten times more abundant than somatic and germ line cells of the human body (Morais et al., 2021). Regarding human health, the gut microbiota contributes to prevent infections caused by pathogens, provide nutrients, participate in metabolism, shape the immune system, and serve as a biological barrier (Haak et al., 2018). In turn, the immune system will affect the microbiota composition (Honda and Littman, 2016). In various disease states, the loss of “health-promoting” bacteria and overgrowth of “disease-promoting” pathogenic bacteria make patients more succumb to sepsis and MODS (Alverdy and Krezalek, 2017). A multi-center study has shown that patients with sepsis have an increased abundance of intestinal microbiota, which is closely related to inflammation caused by Parabacteroides, Clostridium, Bilophila, and other species. Concomitantly, researchers have detected an increased abundance of Enterococcus and other pathogenic bacteria in sepsis patients who died, suggesting that these bacteria may be potential biomarkers for ICU care (Agudelo-Ochoa et al., 2020). It has also been shown that septic patients have a decreased abundance of Faecalibacterium associated with reduced intestinal inflammation (Lankelma et al., 2017). In a single-center case control study of children, the abundance of the following 13 bacteria in septic children were significantly higher than that in the healthy control group: Proteobacteria, Bacilli, Gammaproteobacteria, Enterobacteriales, Pseudomonadales, Lactobacillales, Enterococcaceae, Enterobacteriaceae, Moraxellaceae, Enterococcus, Clostridium innocuum group, Acinetobacter, and Enterococcus durans. Among these bacteria, Enterococcaceae, Enterococcus, and Enterococcus durans showed an increase in their abundance that is positively correlated with the inflammatory indicators CRP and WBC. Furthermore, the abundance of the following seven bacteria was significantly decreased in the guts of septic children: Bifidobacteria, Selenomonas, Aminococcus acidaceae, Daentosaceae UCG-003, Dialister, Dorea longicatena, and *Ruminococcus* sp.5_1_39bFAA. The decrease of intestinal bifidobacteria abundance is negatively correlated to WBC (Liu et al., 2021). The mechanisms behind these shifts in the microbiota composition are unclear (Haak and Wiersinga, 2017). One of the reasons for this phenomenon may be clinical intervention, such as enteral/parenteral feeding, selective oral decontamination/selective decontamination of the digestive tract, as well as the administration of antibiotics, proton pump inhibitors, opioids, catecholamines, and sedatives (Haak and Wiersinga, 2017). In addition, due to the acute phase of inflammatory response and intestinal dysfunction, critically ill patients with sepsis have a greater risk of malnutrition (Liu et al., 2014). Nutritional deficiency is associated with intestinal dysbiosis, characterized as an increase in proteus numbers and a decrease in α-diversity, and it makes epithelial barrier function weaken, which predisposes to bacterial translocation (Ralls et al., 2015; Levesque et al., 2017). And decreased gastrointestinal motility and loss of intestinal epithelial integrity in sepsis patients cause a decrease in anaerobic bacteria (e.g., Lachnospiraceae and Ruminococcaceae), which further impairs the intestinal epithelial function and allows the proliferation of opportunistic pathogenic bacteria (Haak et al., 2018). Fay et al. (2019) examined genetically identical septic C57BL/6 mice from two vendors with different microbiome compositions. Following CLP, significant differences were noted in the mortality and immunophenotype (especially splenic IFN-γ + CD4 + T cells, effector memory CD4 + T cells, central memory CD4 + T cells, and Peyer’s patch effector memory CD4 + T cells). In addition, CLP was performed of naive mice from different suppliers after 3 weeks of cohousing, and the differences in the mortality and immunophenotype disappeared. These findings suggest that the microbiome plays a critical role in the survival of sepsis and the host immune response. This conclusion has also been confirmed by other studies. Depletion of intestinal microbiota with broad-spectrum antibiotics could exacerbate lung and intestinal damage and increase the mortality in sepsis (Xu et al., 2021). Notably, the differential immune responses to sepsis are determined by the microbiome (Cho and Blaser, 2012). A recent study has shown that the lung microbiome is enriched with gut-derived bacteria in murine sepsis and in human patients with ARDS. The presence of gut-specific communities (Bacteroides) is correlated with the intensity of systemic inflammation (Dickson et al., 2016). When the bacterial burden of the gut is minimized, the inflammation and injury sustained with distal organs are lessened during sepsis (Cuevas et al., 1972). Imbalance of the intestinal microbiome regulates the Toll-like receptor 4 (TLR4)/nuclear factor-κB (NF-κB) signaling pathway in the lung immune system, which activates pulmonary oxidative stress and mediates lung injury (Tang et al., 2021). Enhancing the α-diversity of the intestinal flora in mice changes the immune response to sepsis and improves the survival rate of sepsis, which is mediated by the powerful CD4 + T cell response (Fay et al., 2019). The intestinal flora affects not only the cellular immunity but also the humoral immunity. Commensal bacteria directly produce IgA, which has a protective activity against mucosal barrier disruption and the resulting sepsis (Wilmore et al., 2018). These studies indicate that the gut is the predominant motor of sepsis (Klingensmith and Coopersmith, 2016).

#### Role of Gut Microbiota Metabolites on the Sepsis

In recent years, with the developed in-depth understanding of gut microecology, studies have shown that not only gut microbiota itself but also the metabolites of gut microbiota are involved in various functions of the intestinal microenvironment. Bacteria can produce a variety of metabolites, including: SCFAs, vitamins, bile acids, choline metabolites, aromatic compounds, amines, etc., (Van Treuren and Dodd, 2020). In the following sections, we summarized several metabolites affecting the occurrence and development of sepsis.

##### Short-Chain Fatty Acids

Short-chain fatty acids (SCFAs), including butyric acid, acetic acid, and propionic acid, are the most abundant and beneficial metabolites of the intestinal flora (Zhao et al., 2018; Adelman et al., 2020). Several studies have confirmed significantly decreased SCFA concentrations in sepsis patients (Shimizu et al., 2011, 2018; Yamada et al., 2015; Valdes-Duque et al., 2020). Butyric acid reduces the nuclear NF-κB activity, IL-6 and TNF-α levels, and lung tissue neutrophil infiltration by inhibiting the expression of high mobility group protein 1, TLR4, or histone deacetylase to reduce sepsis-related ALI (Li et al., 2018; Liu et al., 2019; Parada Venegas et al., 2019). In addition, acetic acid alleviates septic ALI by regulating the mitogen-activated protein kinase (MAPK) pathway, improving the alveolar permeability, reducing inflammatory factors, and inhibiting oxygen free radical production (Xu et al., 2019). SCFA reverses the progression of sepsis by restoring host immunity and promoting pathogen clearance in an interferon regulatory factor 3-dependent manner (Kim et al., 2020). Moreover, it has been reported that SCFAs contribute to maintain intestinal barrier integrity. SCFAs enhance the immune function of intestinal mucosa by promoting the production of antimicrobial peptides (AMPs; Li et al., 2020). The underlying mechanism may be the promotion of the expression of RegIIIγ and β-defensins 1, 3, and 4 by SCFAs in a GPR43-dependent manner (Zhao et al., 2018). AMPs in the gut, including defensins, cathelicidins, and regenerating gene (Reg)IIIa/b/g, are a class of basic peptides with antibacterial activity secreted by Paneth cells and enterocytes (Zong et al., 2020). Studies have shown that gut-derived AMP deficiency is associated with intestinal barrier failure, leading to bacterial translocation (Zong et al., 2020). In CLP-induced sepsis model mice, cathelicidins gene knockout resulted in increased mortality, impaired intestinal barrier, increased permeability, increased bacterial DNA content of blood, up-regulated expression of intestinal cytokines and inflammatory pathways, and increased M1-type macrophages and neutrophils (Ho et al., 2020).

##### Choline Metabolites

Trimethylamine N-oxide (TMAO) derives primarily from the gut metabolite of choline, carnitine, and phosphatidylcholine (Jiang et al., 2021). Excessive increases in TMAO lead to the release of large amounts of inflammatory mediators that activate the MAPK pathway and the nuclear transcription factor (NF-κB), which mediates vascular inflammation (Kapetanaki et al., 2021). A large number of studies have shown elevated TMAO levels accelerate the progression of inflammatory diseases such as diabetes, atherosclerosis, and heart failure (Gatarek and Kaluzna-Czaplinska, 2021). However, a recent single-center prospective study reveals that TMAO may play different roles in CV and infectious diseases (Chou et al., 2021). A total of 95 patients with sepsis using mechanical ventilation were enrolled in this study and divided into three groups based on TMAO concentration. This study found that plasma TMAO concentration was an independent predictor of successful weaning in mechanically ventilated patients with sepsis after adjustment for APACHE II score and CRP concentration. Septic patients in the lowest TMAO concentrations were at greater risk of non-cardiovascular death and unsuccessful ventilator weaning than were those in higher concentrations. In addition, TMAO concentration was positively correlated with daily energy intake, albumin and prealbumin concentration. These findings suggest that TMAO may be a novel risk biomarker and nutritional indicator for patients with sepsis.

##### Phenolic

One of the degradation products of flavonoids and amino acids by commensal bacteria is desaminotyrosine (DAT; Steed et al., 2017). Flavonoids have anti-inflammatory properties (Peluso et al., 2015). Recently, Wei et al. conducted an experiment that LPS-induced septic mice were intraperitoneally injected with DAT and vehicle control. They found that the survival rate was significantly improved in the DAT-treated group, along with decreased hypothermia and improved clinical scores (Wei et al., 2020). It suggests that DAT modulates systemic immune homeostasis. Nonetheless, whether a flavonoid-enriched diet is a key component to sepsis remains to be studied.

##### Indole Derivatives

Indoles are metabolites of tryptophan metabolism by microbiota (Rattanaphan et al., 2020). It is noteworthy that indole is able to modulate expression of pro-inflammatory genes, increase expression of anti-inflammatory genes and strengthen the epithelial barrier (Nicholson et al., 2012). Researchers found that 5-Hydroxytryptamine (5-HT) significantly increase mortality in a model of sepsis in mice. Furthermore, 5-HT exacerbated the clinical symptoms, and histological damages in the lung, liver, kidney, bowel, and heart (Zhang et al., 2017). Another tryptophan metabolites, indole-3-acetate and indole-3-propionate, was significantly decreased in the both of septic mice and patients, which might result from the gut microbiome disruption in sepsis (Gao et al., 2018; Elmassry et al., 2020). These studies show that Indole derivatives may be a new therapeutic target for sepsis.

##### Vitamin

It is well known that gut microbiota are able to synthesize vitamin K as well as most water-soluble B vitamins such as thiamine (vitamin B1), riboflavin (vitamin B2), nicotinic acid (vitamin B3), pantothenic acid (vitamin B5), pyridoxine (vitamin B6), cobalamin (vitamin B12), biotin (vitamin H), and folates (LeBlanc et al., 2013). The potential biological functions of vitamins include enhancement of immune function, provision of complementary endogenous sources of vitamins, regulation of cell proliferation and so on. In recent years, the efficacy of B vitamins in the treatment of sepsis has received much attention. Hong et al. (2018) has found that prophylactic administration of nicotinamide riboside (NR) can protect lung and heart from injury, and improve the survival rate in sepsis mice, probably via inhibiting HMGB1 release and oxidative stress through the NAD + /SIRT1 signaling. In a rat model of polymicrobial sepsis, vitamin B6 was found to diminish neutrophil infiltration in the both of lung and liver, oxidative markers in the liver and restore catalase activity levels in the lung (Giustina et al., 2019). In addition, vitamin B1 deficiency has been found in patients with critically sepsis (Donnino et al., 2010). Several clinical trials have reported that a combination of cortisol, vitamin C, and Vitamin B1 prevent progressive organ failure, reduce mortality (Marik et al., 2017; Kim et al., 2018) and ICU length of stay (Mitchell et al., 2020) in critically ill patients with septic shock or severe pneumonia. However, an recent single center, retrospective study didn’t find that vitamin B1 and C supplementation improve clinical outcomes (mortality rates, ventilator and ICU-free days) in septic ARDS patients requiring invasive mechanical ventilation (Yoo et al., 2020). Therefore, vitamin may be beneficial to the treatment of sepsis, but much more researches will be needed to verify the clinical benefit of vitamin on patients with septic lung injury.

The above discussion showed that the changes of the intestinal flora and its metabolites can affect the severity of septic ALI/ARDS by regulating the levels of local and systemic inflammation, oxidative cellular stress, and cell infiltration/activation.

### Effect of Sepsis-Related Intestinal Failure on the Lung

**FIGURE 3:**
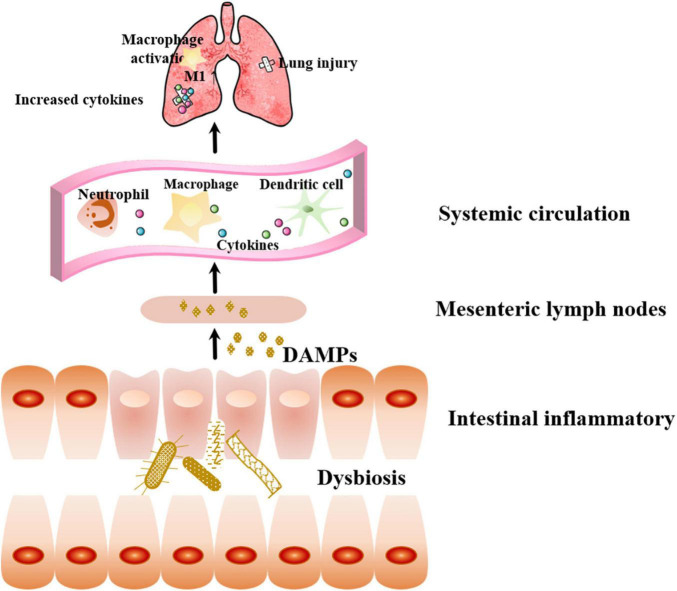
Effect of sepsis-related intestinal failure on the lung. Bacterial dysbiosis can activate local intestinal inflammatory responses, producing DAMPs, which enter the systemic circulation through mesenteric lymphatic vessels. Immune cells recognize DAMPs and secreting pro-inflammatory factors that promote lung injury. DAMPs: danger-associated molecular patterns.

Structural and functional disruption of intestinal barrier integrity in sepsis leads to increased intestinal permeability (Jones and Neish, 2021). Three factors contribute to intestinal barrier damage: visceral hypoperfusion or ischemia, restoration of intestinal blood flow during resuscitation leading to ischemia-reperfusion injury, and loss of the intestinal barrier function, which allows bacteria, endotoxins, or both to cross the mucosal barrier (Otani and Coopersmith, 2019). Bacterial translocation can activate local intestinal inflammatory responses, producing DAMPs, which enter the systemic circulation through mesenteric lymphatic vessels. DAMPS are recognized by cells expressing intrinsic pattern receptors in the intrinsic immune system, including macrophages, white blood cells, and dendritic cells, then secreting pro-inflammatory factors that promote lung injury and the development of MODS, which further aggravate intestinal barrier injury and lead to a vicious cycle (Pugin and Chevrolet, 1991; Figure 3).

Macrophages, commonly polarized into M1 (pro-inflammatory) and M2 (anti-inflammatory) types according to responses to environmental stimuli, are key orchestrators in the pathogenesis of ALI/ARDS (Shapouri-Moghaddam et al., 2018). In the acute phase of sepsis, macrophages polarize into the M1 phenotype to activate TLRs or other recognition receptors due to impaired intestinal barrier function, bacterial translocation, and increased inflammation (Osterberg et al., 1997), releasing a variety of inflammatory cytokines, such as IL-1β, IL-6, and TNF-α. Proinflammatory factors induce the recruitment of neutrophils in the blood circulation, then migrate to the lungs and alveolar cavities, resulting in lung injury. Accumulating evidence indicates that some natural or synthetic materials can ameliorate the prognosis of sepsis-induced ALI in animal models by inhibiting pulmonary M1 polarization and altering the macrophage function (Bittencourt-Mernak et al., 2017; Pinheiro et al., 2017; Wang et al., 2018, 2019; Zhuo et al., 2019). Thus, the regulation of macrophage polarization from M1 to M2 may be a novel therapeutic strategy of ALI/ARDS (Chen et al., 2020).

### Prospect

As the intestinal barrier plays an important role in sepsis-induced lung injury, targeted microbiota has the potential for prevention and treatment of lung injury. Fecal microbiota transplantation (FMT) can help the recipient to establish a normal intestinal microecological environment and may be considered a useful therapy for sepsis in the future. In the last few years, FMT has made great strides in correcting microbiota disorders, repairing the intestinal barrier, and regulating immunity (Ooijevaar et al., 2019). In the intervention of FMT in lipopolysaccharide (LPS)-induced ALI, anti-inflammatory and antioxidant mechanisms may play an important role. FMT intervention could correct the changes in the intestinal flora and improve lung injury by inhibiting the activation of the PI3K/AKT/NF-κB signaling pathway and decreasing the expression of intercellular cell adhesion molecule-1 (ICAM-1; Yin et al., 2019). In addition, FMT significantly reduces the TNF-α, IL-1β, and IL-6 levels as well as inflammatory cell infiltration and interstitial exudate, thereby improving LPS-induced endotoxic ALI in rats, which is associated with the decreased expression of TGF-β1, Smad3, and P-ERK (Li et al., 2020). In the LPS-induced ALI mouse model of intestinal microbiota imbalance pretreated with antibiotics, an increased diversity of the intestinal flora and abundance of beneficial bacteria producing SCFAs that antagonize acute lung injury was observed by reconstructing the intestinal flora through FMT. This inhibits the activation of the TLR4/NF-κB signaling pathway in the lung, inflammation, and the release of oxidative stress factors in ALI animals (Tang et al., 2021). Therefore, FMT therapy can be used in the treatment of lung disorder during sepsis. However, existing research in this field remains limited to animal experiments. More basic trials are needed to clarify the mechanisms of FMT in lung injury, as well as large clinical trials to evaluate the efficacy and safety of FMT therapy.

## Conclusion

Many aspects of lung injury in sepsis have not been thoroughly studied, and the interaction between intestine and lung in sepsis is still a promising research direction. During septic lung injury, the expression of intestinal tight junction protein, the activity of MLCK, and the regulation of IECs proliferation and apoptosis are altered by cytokine storm, leading to gut hyperpermeability. Increase of the intestinal permeability leads to the translocation of gut microbiota, resulting in intestinal inflammation and a cascade of inflammatory reactions driving acute lung injury. Moreover, lung dysbiosis in sepsis-induced ALI/ARDS may cause gut dysbiosis. And the changes of the intestinal flora and reduction of beneficial metabolites in sepsis promote lung injury exacerbation by regulating local and systemic inflammation. Persistent inflammation leads to devastating consequences. Theoretically, therapies that restore the intestinal integrity, microbiome, and homeostasis balance between the two systems through FMT are efficient, but so far basic research and clinical trials are not sufficient. An in-depth understanding of gut–lung crosstalk may provide clues for the regulation of homeostasis in sepsis and contribute to the development of effective therapies to prevent sepsis-induced ALI/ARDS.

## Author Contributions

XZ developed the manuscript and figures. YL contributed to proofreading and revising. Both authors contributed to the article and approved the submitted version.

## Conflict of Interest

The authors declare that the research was conducted in the absence of any commercial or financial relationships that could be construed as a potential conflict of interest.

## Publisher’s Note

All claims expressed in this article are solely those of the authors and do not necessarily represent those of their affiliated organizations, or those of the publisher, the editors and the reviewers. Any product that may be evaluated in this article, or claim that may be made by its manufacturer, is not guaranteed or endorsed by the publisher.
